# Identification and Validation of TRIM25 as a Glucose Metabolism Regulator in Prostate Cancer

**DOI:** 10.3390/ijms23169325

**Published:** 2022-08-19

**Authors:** Chao Li, Peng Dou, Xin Lu, Pengwei Guan, Zhikun Lin, Yanyan Zhou, Xin Lu, Xiaohui Lin, Guowang Xu

**Affiliations:** 1 School of Computer Science and Technology, Dalian University of Technology, Dalian 116024, China; 2 CAS Key Laboratory of Separation Science for Analytical Chemistry, Dalian Institute of Chemical Physics, Chinese Academy of Sciences, Dalian 116023, China; 3 Liaoning Province Key Laboratory of Metabolomics, Dalian 116023, China

**Keywords:** prostate cancer, glucose metabolism, tumor malignancy, weighted differential network, TRIM25

## Abstract

Prostate cancer (PCa) malignant progression is accompanied with the reprogramming of glucose metabolism. However, the genes involved in the regulation of glucose metabolism in PCa are not fully understood. Here, we propose a new method, DMRG, which constructs a weighted differential network (W-K-DN) to define the important metabolism-related genes. Based on biological knowledge and prostate cancer transcriptome data, a tripartite motif-containing 25 (TRIM25) was defined using DMRG; TRIM25 was involved in the regulation of glucose metabolism, which was verified by overexpressing or knocking down TRIM25 in PCa cell lines. Differential expression analysis of TCA cycle enzymes revealed that TRIM25 regulated isocitrate dehydrogenase 1 (IDH1) and fumarate hydratase (FH) expression. Moreover, a protein–RNA interaction network of TRIM25 revealed that TRIM25 interacted with RNA-binding proteins, including DExH-box helicase 9 and DEAD-box helicase 5, to play a role in regulating the RNA processing of metabolic enzymes, including IDH1 and FH. Furthermore, TRIM25 expression level was found to be positively correlated with Gleason scores in PCa patient tissues. In conclusion, this study provides a new method to define genes influencing tumor progression, and sheds light on the role of the defined TRIM25 in regulating glucose metabolism and promoting PCa malignancy.

## 1. Introduction

Prostate cancer (PCa) is a malignant tumor that originates from the prostate epithelial cells. Compared with the advanced stages, the most obvious distinguishing feature of early-stage PCa is the functionality of glucose metabolism. Early-stage PCa consumption of citric acid fuels oxidative phosphorylation to restore the TCA cycle function. However, advanced castration-resistant prostate cancer (CRPC) is associated with a higher level of glycolysis and lactic acid compared to early-stage PCa (androgen-dependent PCa) [1]. Lactic acid serves as a raw material for energy production and further catabolism [2], and contributes to an acidic microenvironment, thus mediating tumor reprogramming [3]. Targeting the lactic acid transporter MCT4 to reduce lactic acid secretion helps the treatment of CRPC [4]. Therefore, targeting glucose metabolism is now a new direction in the treatment of tumors, and the inhibition of proteins involved in regulating glucose metabolism can help to prevent malignant progression [5].

Glucose metabolic reprogramming promotes cancer progression, which is regulated by the expression of associated genes. Given the difficulty in finding genes that regulate the reprogramming of glucose metabolism from a large number of genes, multi-omics integration analysis helps to define key genes that lead to metabolic reprogramming in PCa. To identify the important biomolecules, several computational methods based on network analysis have been proposed [6,7,8]. The differential network (DN) analysis method aims at identifying the disease-related important biomolecules or modules [9]. DN can be classified into two categories: statistics-based differential network (S-DN) and knowledge-based differential network (K-DN). S-DN is constructed by statistical measures, such as Pearson correlation coefficient (PCC), partial correlation coefficient, and mutual information (MI). For example, PB-DSN used PCC to measure the feature relationship and construct the differential subnetwork for identifying the potential biomarkers of hepatocellular carcinoma [10]. In contrast, K-DN enables the use of known biological knowledge (protein interactions, reactions, pathways, etc.) to identify relationships between biomolecules, reduce false associations, and guide biological network construction. Combining gene expression data and protein–protein interaction (PPI) networks to define key nodes of biological development (MarkRank) has shown good predictive accuracy and high specificity associated with the disease [8]. K-DN can also cover experimental data gaps to explore the key factors affecting disease. Therefore, combining the advantages of the two approaches and the metabolic background knowledge will help to define the key metabolism-related genes affecting the progression of PCa.

Several TRIM-associated proteins are involved in the regulation of glucose metabolism, and play a key role in driving tumor development. TRIM21 regulates the pentose phosphate pathway and controls glucose metabolism during epithelial mesenchymal transition to provide sufficient energy for tumor metastasis [11,12]. In PCa, TRIM25 promotes cell proliferation and chemotherapy resistance [13], which can indirectly stabilize C-myc to enhance tumor development [14]. Moreover, C-myc is a metabolic regulator in regulating glycolysis and lipid synthesis [15,16]. Although the above studies suggest a potential role for TRIM25 in the regulation of glucose metabolism, there are no studies showing that TRIM25 regulates glucose metabolism to promote the malignant progression of tumors.

In this study, we propose a novel network analysis method (DMRG) for defining the important metabolism-related genes. Based on DMRG, TRIM25 was defined as a metabolism regulator affecting the malignant progression of PCa. It was found that TRIM25 leads to a decrease in TCA metabolite levels and an increase in lactic acid levels. Subsequently, the expression levels of glycolytic enzymes and TCA cycle enzymes were further analyzed. It was found that TRIM25 downregulated IDH1 and FH expression. Protein profiling combined with a network approach revealed that TRIM25 interacts with DExH-box helicase 9 (DHX9) and DEAD-box helicase 5 (DDX5) to regulate the RNA of IDH1. Furthermore, immunohistochemical analysis of PCa tissue microarrays revealed that increased expression of TRIM25 correlated with the degree of PCa malignancy.

## 2. Results

### 2.1. DMRG Method and Defining of Potential Key Genes

**Figure 1:**
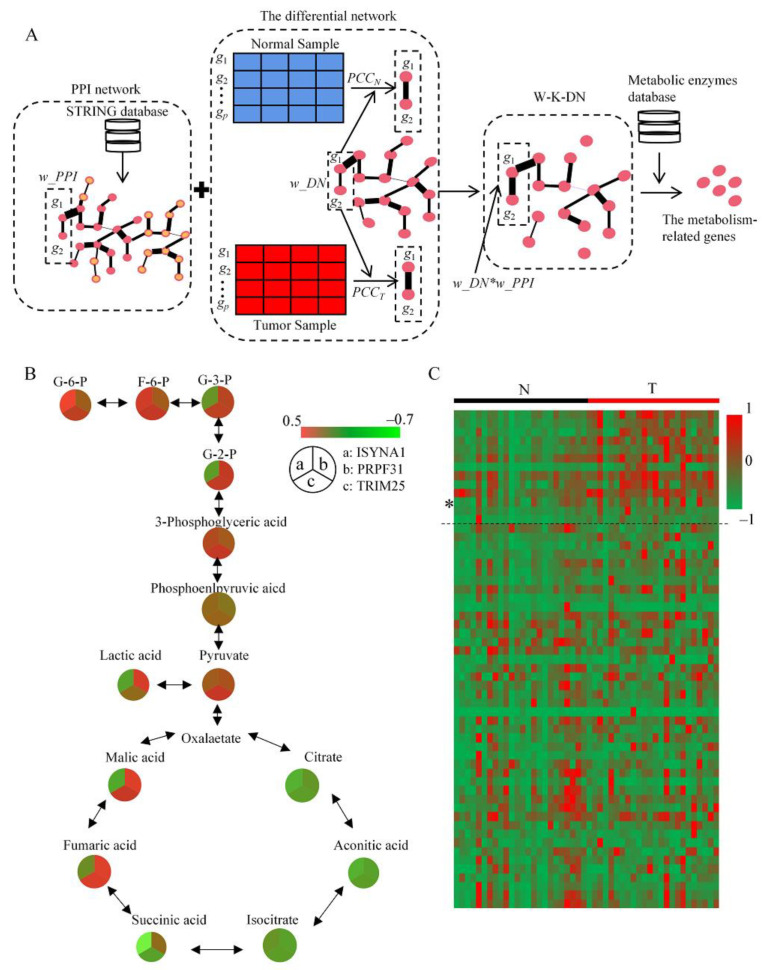
Screening of the genes that promote prostate cancer development. (**A**) Flowchart of the method (DMRG) of defining the important metabolism-related genes. (**B**) Analysis of the correlation between three key genes (ISYNA1, PRPF31, TRIM25) and glucose metabolism. (**C**) Heat map analysis of TRIM family gene expression levels in 24 paired PCa/normal tissues from the transcriptomic dataset. “N” represents normal tissue. “T” represents PCa tumor tissue. * *p* < 0.05.

To define the important metabolism-related genes, we proposed a novel network analysis method, DMRG. The flowchart of DMRG is shown in Figure 1A. First, the PPI network was constructed by STRING database (STRING V11.0, accessed on 14 July 2020, https://www.string-db.org/). Then, the differential network was constructed by Spearman’s correlation coefficient (SPCC) based on PCa transcriptome data, which contained 24 paired PCa/normal tissues. Next, combining the PPI network and the differential network, the W-K-DN, a weighted knowledge-based differential network, was built. Finally, the network node importance score, which was defined by the topology properties of the W-K-DN, and the metabolic enzymes database were used to identify the metabolism-related genes. Table 1 gives the top 10 ranked genes based on the network node importance score and the metabolic enzymes database. They play important roles in cancer, for example, MYC, PETN, and CD44 have been widely reported, and the nodal RANBP2 has been identified as a biomarker in PCa [17].

In addition to the topological structure analysis of the W-K-DN, the area under the receiver operating characteristic curve (AUC) was also used to confirm the important genes. Table 1 shows that node RANBP2 had the highest AUC value in all the top-ranked molecules. Hence, we focused on node RANBP2 and its subnetwork. The subnetwork of RANBP2 is composed of the nodes and edges adjacent to RANBP2 in the W-K-DN. There are 364 genes adjacent to RANBP2, including 60 differential expression genes and 112 PCa-related genes. Twenty-two genes that were differentially expressed and related to PCa were selected for survival analysis with the TCGA PCa dataset (Supplementary Table S1). Among these genes, PRPF31, TRIM25, and ISYNA1 were highly expressed in PCa, and patients with relatively higher levels of these genes had shorter survival (Supplementary Figure S1).

By combining metabolomics data with the transcriptome from the same tissues, we further analyzed the correlation between the above gene expression levels and glucose metabolism levels. Generally, the expression levels of PRPF31, TRIM25, and ISYNA1 showed a positive correlation with glycolytic metabolites, and a negative correlation with TCA cycle metabolites (Figure 1B). PRPF31 is involved in the regulation of the metformin response to type 2 diabetes [18], which suggests that PRPF31 has a potential glucose metabolism function, but there is no evidence that it affects cancer development; in contrast, ISYNA1 is responsible for inositol synthesis [19], but its effect on glucose metabolism is more indirect. Several TRIM-associated proteins are involved in the regulation of glucose metabolism, and play a crucial role in driving tumor development [11,12]. Therefore, TRIM-associated protein expressions were analyzed based on PCa transcriptome data, and the expression levels of several TRIM-associated proteins (above the dashed line), including TRIM25 (*), in tumor tissues were significantly higher than those in the normal tissues (Figure 1C and Supplementary Table S2). The above results indicate that TRIM25 is associated with the regulation of glucose metabolism in PCa.

### 2.2. TRIM25 Promotes Glycolysis to Mediate Cell Growth

**Figure 2:**
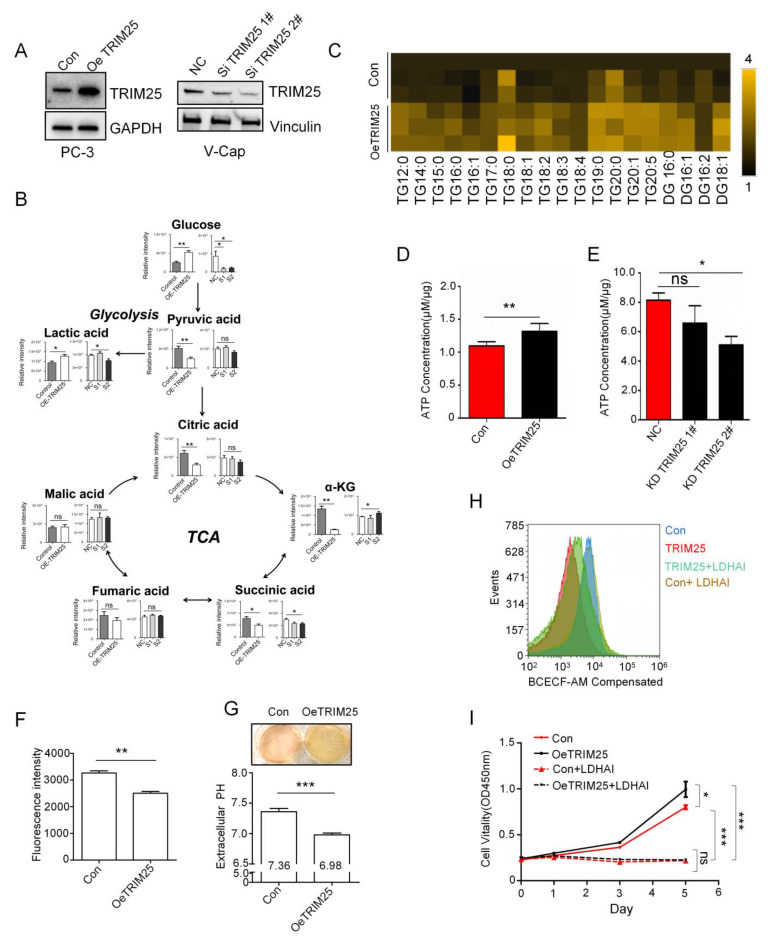
TRIM25 promotes glycolysis and upregulates cellular lactic acid secretion. (**A**) TRIM25 protein level in stable overexpression PC3 and RNAi knockdown V-Cap cells. (**B**) GC–MS detected metabolites of glycolytic and TCA cycle in TRIM25 overexpression PC3 and knockdown V-Cap cells. NC, negative control; S1, SiTRIM25 1#; S2, SiTRIM25 2#. (**C**) Heat map showing lipid level in TRIM25 overexpression PC3 cells. (**D**,**E**) Analysis of ATP levels in TRIM25 overexpression PC3 and knockdown V-Cap cells. *, *p* < 0.05; **, *p* < 0.01, as identified by *t*-test. (**F**) BCECF-AM probe detects intracellular pH changes in TRIM25 overexpression PC3 cells. **, *p* < 0.01, as determined by unpaired *t*-tests. (**G**) Culture medium pH change in TRIM25 overexpression PC3 cells. (**H**) Flow cytometry analysis of fluorescence intensity of BCECF-AM labeled TRIM25 overexpression PC3 cells under 10 μM LDH-IN treatment. (**I**) CCK8 analysis of cell growth in TRIM25 overexpression PC3 cells under 20 μM LDH-IN treatment. Error bars indicate the mean ± SD of at least three independent replicates. ns, *p* > 0.05; *, *p* < 0.05; ***, *p* < 0.001, as identified by ANOVA.

To further analyze the roles of TRIM25 in the regulation of glucose metabolism, TRIM25 overexpression or knockdown cells were constructed (Figure 2A). Higher levels of glucose and lactic acid were observed in the TRIM25 overexpression group than in the control group, while most of the metabolites were reduced in the TCA cycle, such as citric acid, α-KG, and succinic acid (Figure 2B). In contrast, TRIM25 knockdown V-Cap cells had relatively lower levels of glucose and lactic acid. For TCA cycle metabolites, α-KG and succinic acid were significantly increased and decreased in the knockdown group, respectively. Other metabolites were not significantly different (*p* > 0.05) compared with the control group (Figure 2B). Interestingly, the total pyruvic acid level was significantly reduced in overexpressed TRIM25 cells. Pyruvic acid can be converted to acetyl coenzyme A to enter the lipid metabolism pathway. Therefore, intracellular lipids were examined. Triglyceride (TG) and diglyceride (DG) were significantly elevated with regards to TRIM25 overexpression (Figure 2C). In addition, overexpression of TRIM25 produced more ATP (Figure 2D), while knockdown of TRIM25 reduced intracellular ATP (Figure 2E). The above results suggest that TRIM25 regulates the reprogramming of glucose metabolism, promotes lipid synthesis, and produces more ATP to meet the needs of cell proliferation.

To clarify the effect of TRIM25 on intracellular pH as well as on the extracellular microenvironment, we analyzed the intracellular pH using a BCECF-AM probe. Compared to the control group, overexpressed TRIM25 had a relatively low fluorescence intensity (Figure 2F). This suggests that pH was decreased in cells. Interestingly, the cell culture medium of the TRIM25 overexpression group became yellow (Figure 2G), indicating an acidic environment. 

Cell culture medium pH was detected by pH meter, and the results show that the pH (6.98) of the TRIM25 overexpression group was markedly (*p* < 0.001) lower than that of the control group (pH = 7.36) (Figure 2G). This indicates that TRIM25 promoted the production of lactic acid and its further secretion into the culture medium. To clarify that TRIM25 drives intracellular pH changes by regulating lactic acid, intracellular pH was further analyzed by blocking the lactic acid synthesis pathway with an LDH inhibitor (LDH-IN) (Supplementary Figure S2A), and it was found that 20 μM LDH-IN was capable of blocking the conversion of pyruvic acid to lactic acid (Supplementary Figure S2B). TRIM25 overexpression promoted cell acidification (Figure 2H) and cell proliferation (Figure 2I). Interestingly, blocking the lactic acid synthesis pathway significantly reversed the decrease in intracellular pH caused by TRIM25, and the cell proliferation promoted by TRIM25 was significantly inhibited. Moreover, there was no proliferation difference between the control (Con) group and TRIM25 overexpression group under complete blockade of the glycolytic pathway (Figure 2I); however, the TRIM25 overexpression group still had a lower pH than the control group (Figure 2H). Therefore, the effect of TRIM25 on cellular pH is not completely brought about by the regulation of cell proliferation. The above results suggest that high levels of TRIM25 exhibit strong glycolysis and high-level lactic acid production in PCa cells.

### 2.3. TRIM25 Remodels TCA to Enhance Glycolysis Flux

**Figure 3:**
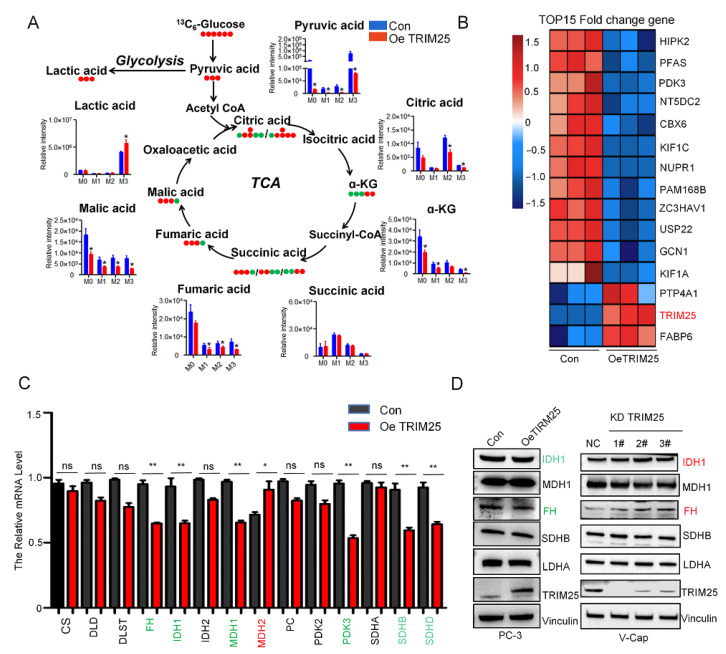
TRIM25 enhances glycolytic flux by downregulating the expression level of TCA cycle-related enzymes. (**A**) GC–MS detection of metabolic flux changes in glucose-^13^C6-labeled TRIM25 overexpression PC3 cells. M0, unlabeled mass of isotopologue; Mn, native metabolite mass (M) plus number of isotopically labeled carbons (n). Error bars show the mean ± SEM of three independent replicates or more. ns, not significant; *, *p* < 0.05; **, *p* < 0.01, as determined by unpaired *t*-tests. (**B**) Heat map plot with the top 15 most significant differential genes in transcriptomics. (**C**) The mRNA expression of TCA-related genes in TRIM25 overexpression PCa cells. *, *p* < 0.05; **, *p* < 0.01, as determined by unpaired *t*-tests; ns, not significant. (**D**) The protein expression of TCA-related enzymes in PCa cells.

To clarify the effect of TRIM25 on metabolic flux in glucose metabolism, [U-^13^C]glucose tracing was used to profile the flux change of glycolysis and TCA. M0 represents no ^13^C labeling, while M1 stands for one ^13^C atom, and so forth. One molecule of [U-^13^C]glucose is converted by glycolysis into two molecules of M3 pyruvic acid; M3 pyruvic acid is converted to M3 lactic acid. As shown in Figure 3A, M3 lactic acid was significantly (*p* < 0.05) increased, while M2 citric acid, fumaric acid, and malic acid were reduced (Figure 3A). M3 metabolites such as malic acid, fumaric acid, α-KG, and citric acid were reduced, which indicates that the reverse TCA cycle was reduced. This suggests that TRIM25 increases glycolytic flux by changing TCA flux.

To analyze the causes of metabolic flux changes, we analyzed TRIM25-regulated gene expression by the transcriptome. The heat map in Figure 3 shows the top 15 different expression genes in TRIM25 overexpression cells. The expressions of metabolism-related genes FABP6, PDK3, and PFAS were significantly changed (Figure 3B). Further analysis of TCA-related gene expression showed that FH, IDH1, PDK3, and other genes were significantly downregulated (green marked), while MDH2 was significantly upregulated (red marked) (Figure 3C). The protein levels of the genes were further detected and the result showed that FH and IDH1 were downregulated with TRIM25 overexpression, while FH and IDH1 were upregulated with TRIM25 knockdown (Figure 3D). Therefore, it can be concluded that TRIM25 regulates TCA-related enzyme expression involved in the regulation of TCA metabolic fluxes. 

### 2.4. TRIM25 Is Involved in RNA Processing to Regulate the Expression of Metabolic Enzymes

**Figure 4:**
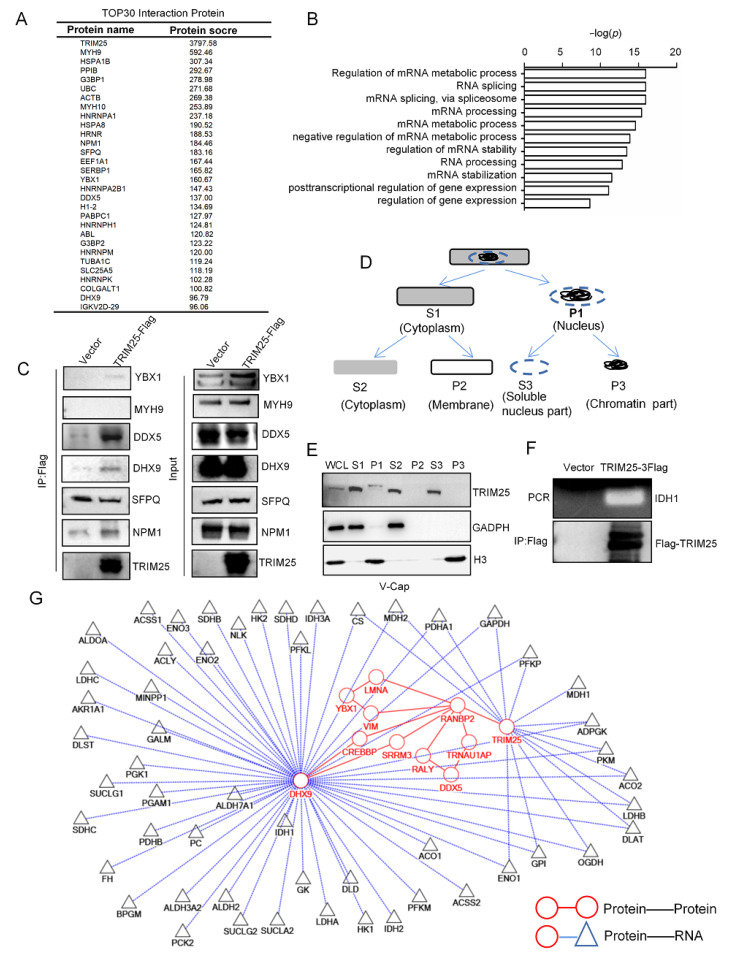
TRIM25 performs RNA processing functions. (**A**) Top 30 proteins based on protein scoring by LC-MS. (**B**) GO annotated TRIM25-interacting protein function. (**C**) Verification of TRIM25-interacting proteins by immunoprecipitation with Flag-M2 beads. (**D**) Flowchart of cell fractionation. (**E**) TRIM25 expression in various fractions of V-Cap cells. (**F**) RNA-Chip validated TRIM25 binding to RNA of IDH1. (**G**) The subnetwork of TRIM25 constructed by integrating the W-K-DN and the protein–RNA interactions.

To explore the potential molecular mechanism of TCA cycle regulation of TRIM25, TRIM25-interacting proteins were analyzed. The top 30 interacting proteins were obtained based on a protein profile scoring screen. TRIM25 is well known for its E3 ubiquitin ligase function, ubiquitin C (UBC) would be expected to be present in Figure 4A. However, there were no glucose metabolism-related enzymes among the top 30 proteins, so the metabolic regulatory role of TRIM25 through its E3 ubiquitin ligase function will not be considered. 

The RNA processing function of TRIM25 was further explored. The TRIM25-interacting proteins were defined by RNA-binding proteins such as DDX5 and DHX9 (Figure 4A). Subsequently, GO annotation of TRIM25-interacting proteins revealed that TRIM25-interacting protein functions were mainly enriched in RNA processing and RNA metabolism (Figure 4B). Validation of TRIM25-interacting proteins revealed that TRIM25 interacted with YBX1, DDX5, and DHX9 (Figure 4C), and the TRIM25 truncator was further analyzed for the interaction with DDX5, and the TRIM25 PS domain (PRY/SPRY domain with RNA-bind activity) was found to be more strongly bound to DDX5 (Supplementary Figure S3). 

RNA processing tends to occur in the nucleoplasmic region of the cell [20], so we detected the cellular localization of TRIM25 by cell fractionation (Figure 4D) and found that TRIM25 was expressed in the cytoplasm and nucleoplasmic region, but was not detected in the chromatin (Figure 4E). RNA-Chip experiments showed that TRIM25 bound to the RNA of IDH1 (Figure 4F). Research has reported the RNA-binding activity of TRIM25 and the TRIM25-binding RNA by CLIP-seq [21]. To initially explore the proteins that co-regulate metabolism with TRIM25, the subnetwork of TRIM25 was constructed by integrating the W-K-DN and the protein–RNA interactions from StarBase [22]. As shown in Figure 4G, circular nodes vs. circular nodes indicated protein–protein interactions, and circular nodes vs. triangular nodes indicated protein–RNA interactions. It was found that the TRIM25-binding RNA contained a variety of metabolism-related RNAs, moreover, DHX9 interacting with TRIM25 bound RNA of IDH1 and FH. Therefore, this result suggests that TRIM25 exerts an RNA processing function of regulating IDH1 expression to influence the TCA cycle.

### 2.5. TRIM25 Expression Level Is Correlated with the Malignance of PCa

**Figure 5:**
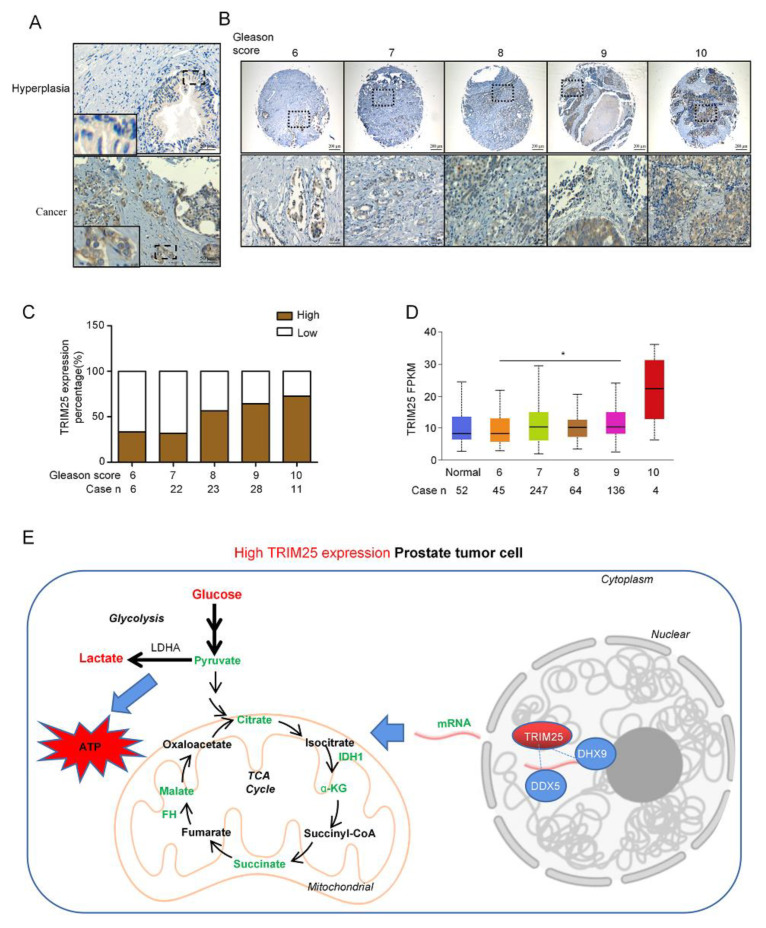
TRIM25 is associated with prostate cancer malignancy. (**A**) TRIM25 protein levels in prostate hyperplasia tissue and prostate cancer by immunohistochemistry. Scale bar = 50 μm. (**B**) Images of immunohistochemical staining of TRIM25 protein levels in prostate cancer with different Gleason scores. Scale bar = 200 μm, Zoom Scale bar = 50 μm. (**C**) Statistical analysis of the proportion of TRIM25 expression in prostate cancer tissues with different Gleason scores. (**D**) Analysis of TRIM25 expression levels in PCa with different Gleason scores from the TCGA database. (**E**) Working model of high TRIM25 levels promoting prostate cancer malignancy. * *p* < 0.05.

To clarify whether TRIM25 is associated with the malignancy of PCa, TRIM25 protein levels were detected in a PCa tissue microarray through immunohistochemistry (IHC) staining. IHC staining indicated that TRIM25 was highly expressed in PCa cells, and mainly located in cytoplasm and nuclear region (Figure 5A). To explore the role of TRIM25 in the malignance of PCa, we analyzed TRIM25 expression with different Gleason stages, IHC staining suggested that TRIM25 levels were markedly increased in PCa patients with high Gleason scores (Figure 5B). Additionally, 72.7% (8/11) of the Gleason 10 PCa tissues had a higher IHC score, 64.3% (18/28) of the Gleason 9 PCa tissues, 56.5% (13/23) of the Gleason 8 PCa tissues had a higher IHC score, 31.8% (7/22) and 33.3% (2/6) of Gleason 7 and 6 PCa tissues had high TRIM25 IHC score (Figure 5C). Moreover, TRIM25 mRNA expression level in PCa cancer patients was analyzed with the Gleason score, which was obtained from the TCGA dataset (http://ualcan.path.uab.edu, accessed on 1 January 2020), and the expression level of TRIM25 was increased with Gleason score (Figure 5D); TRIM25 expression level was significantly higher in Gleason 9 than that in Gleason 6 (*p* < 0.05). 

Moreover, PCa patients with higher TRIM25 expression had lower disease-free survival (DSF) times (Supplement Figure S1). Collectively, TRIM25 binds with DHX9 and DDX5 to participate in RNA processing to downregulate TCA enzyme expression, thereby remodeling the TCA cycle and glycolytic flux is increased to promote tumor proliferation (Figure 5E).

## 3. Discussion

PCa is a typical metabolic disease in which development and metabolic reprogramming are poorly understood. To identify important genes involved in PCa progression, a new method DMRG is proposed. DMRG constructs the weighted differential network by integrating PPI database and prostate cancer transcriptome data to identify important genes by node importance score and the metabolic enzymes database. The process of DMRG includes four parts: construction of PPI network based on protein–protein interactions, construction of differential network based on Spearman’s correlation coefficient, construction of the weighted differential network (W-K-DN) by the combination of PPI network and differential network, and identification of the important genes based on the W-K-DN and the metabolic enzymes database. The DMRG method is advantageous in combining statistics data and biological knowledge information to ensure the accuracy and specificity of biological networks. Meanwhile, the edge weights in the W-K-DN contain two parts: the associated confidence score from PPI database and differential association from specific disease data. The key genes defined by DMRG are correlated with the metabolic enzymes database to determine the TRIM25 glucose metabolism regulatory function. TRIM25 is considered as a type of oncoprotein that plays a regulatory role in reactive oxygen balance; it also enhances tumor cell proliferation and drug resistance by promoting Keap1 degradation [23]. Interestingly, in PCa, highly expressed TRIM25 promotes cell proliferation and attenuates docetaxel-induced apoptosis [13]. 

We found that TRIM25 expression levels show a positive correlation with the levels of glycolysis, and a negative correlation with the levels of TCA cycle metabolites, and TRIM25 shows a positive correlation with the Gleason scores of PCa samples. This suggests that TRIM25 promotes malignancy in prostate cancer through the metabolic remodeling of glucose metabolism. Subsequently, cellular validation revealed that high expression of TRIM25 accelerated glucose uptake and enhanced glycolysis to produce large amounts of lactic acid, while low expression of TRIM25 indicated the opposite metabolic trend. In addition, PCa cells with high TRIM25 expression and an acidic culture medium suggests that a large amount of lactic acid was secreted into the culture medium. The high level of lactic acid altered the tumor microenvironment and promoted tumor migration [24,25]. Lactic acid has the ability to regulate tumor reprogramming [26], and suppress tumor immunity [27]. Thus, TRIM25 promotes the production of lactic acid to play an important function in regulating the malignant transformation of PCa. Moreover, high expression of TRIM25 promotes lipid synthesis, and abnormal lipid metabolism also contributes to the malignant progression of PCa [28,29].

TRIM25, as a protein with both E3 ubiquitin ligase and RNA-binding function, may have multiple mechanisms to regulate the TCA cycle. TRIM25 has three domains: the RING, coiled-coil (CC), and PRY/SPRY (PS) domains. The RING domain supports protein ubiquitination and degradation, the PS domain supports RNA binding activity. We found that TRIM25 interacted with a variety of RNA-binding proteins and participated in RNA processing functions, it was expressed in the cytoplasmic as well as nucleoplasmic regions of cells, and the nucleoplasmic region of cells is the place for RNA processing [20]. Moreover, the C-terminus (PS domain) of TRIM25 with RNA binding activity can bind to viral RNA and activate the NF-κB signaling pathway to resist viral invasion [30]. TRIM25 binds to caspase-2 mRNA and downregulates caspase-2 protein levels to mediate drug insensitivity in colon carcinoma cells [31]. Moreover, we investigated the reported fact that TRIM25 binds to intracellular RNA [21], and verified that TRIM25 bound RNA of IDH1 by RNA-Chip experiments. In our investigation on the regulation of TRIM25 to IDH1, we verified two RNA-binding proteins, DHX9 and DDX5, that interact with TRIM25. DHX9 prevents the formation of RNA advanced structures, such as RNA–RNA hybrid strands, RNA–DNA–RNA hybrid strands (known as the R-loop), and other RNA advanced structures, to prevent abnormal termination of transcription [32,33]. DDX5 possesses an RNA advanced structure unwinding characteristic, and also an mRNA export function [34]. We found that TRIM25 binds DDX5 through the CC and PS domains; therefore, we speculate that TRIM25 may regulate DDX5 to affect RNA transport. Further exploration into the molecular mechanisms of TRIM25 regulation of metabolic pathways is to be performed in the future.

In conclusion, our study developed a method, DMRG, to define important genes, and, combined with prostate cancer transcriptome data to define TRIM25, we clarified the role of TRIM25 in glucose metabolic reprogramming. The results provide a scientific basis for TRIM25 as a potential clinical therapeutic target for PCa.

## 4. Materials and Methods

### 4.1. DMRG Method 

First, DMRG constructed the PPI network. It obtained PPIs from the STRING database (STRING V11.0, accessed on 14 July 2020, https://www.string-db.org/), which is one of the most commonly used PPI databases and contains 19,566 human proteins and 11,759,454 functional interactions. If two proteins contribute jointly to a biological function, they set a link. Hence, the PPI network was constructed by protein–protein interactions from STRING. Meanwhile, each link in the STRING database was given an associated confidence score, which represents the reliability of the association based on the evidence. The associated confidence score was discretized by quantile discretization and used as the edge weight (*w_PPI*) of the PPI network. 

Then, DMRG adopted Spearman’s correlation coefficient (SPCC) to construct the DN based on the specific disease gene expression data. For each gene pair (*g*_1_, *g*_2_), the SPCC in each condition (tumor, normal) was computed, *PCC_T_*(*g*_1_, *g*_2_) represented the SPCC in the disease state and *PCC_N_*(*g*_1_, *g*_2_) represented the SPCC in the normal state. DMRG established an edge between *g*_1_ and *g*_2_ in DN if their correlation difference between the two states was great than 0.8, and the edge weight (*w_DN*(*g*_1_, *g*_2_)) of the DN was determined as follows:

(1) $$w\_DN(g_{1},g_{2})=\left\{ \begin{array}{l} \Delta PCC(g_{1},g_{2}),\;if\;\Delta PCC(g_{1},g_{2})>0.8\\ 0\;\;\;\;\;,\mathrm{otherwise} \end{array}\right.$$

where △*PCC*(*g*_1_, *g*_2_) = |*PCC_T_* − *PCC_N_*|. 

DMRG fused the PPI network and the DN to generate the weighted differential network W-K-DN, where the nodes are genes from the specific disease gene expression data. The weights of edges were defined as w_DN*w_PPI.

Finally, DMRG performed network topology analysis to identify the important genes. The edge information in the W-K-DN reflects the changes in node associations between disease and normal states. Based on the edge weights in the W-K-DN, degree centrality was used to evaluate the genes. Let *v* be a node in the W-K-DN and *neighbor*(*v*) be the set of the nodes adjacent to node *v,* then node importance score *s*(*v*) is defined as the summation of the edge weights adjacent to node *v* (see Formula (2)):

(2) $$s(v)=\sum_{u\in nieghbor\left(v\right)}^{}w\_DN\left(u,v\right)*w\_PPI\left(u,v\right)$$

### 4.2. Cell Culture 

HEK-293T, V-Cap, and PC3 cells (purchased from ATCC) were cultured in Dulbecco’s Modified Eagle Medium (DMEM, Gibco, Waltham, MA, USA) supplemented with 1% penicillin–streptomycin solution (Gibco, Waltham, MA, USA) and 10% fetal bovine serum (Biological Industries, Beit HaEmek, Israel) in a humidified incubator at 37 °C with 5% CO_2_.

### 4.3. Western Blotting 

Proteins were extracted from the cell using RIPA lysis buffer with protease inhibitor cocktail and phosphatase inhibitor cocktail (MCE, Shanghai, China), then transferred onto PVDF membrane to perform SDS-PAGE (Millipore, Billerica, MA, USA). Next, the PVDF membrane was incubated in phosphate buffer saline (PBST) containing 0.05% Tween-20 and 5% non-fat milk for 1 h at room temperature. Primary antibodies were incubated overnight at 4 °C, and secondary antibodies (1:10,000, proteintech) for 1 h at room temperature. Blots were developed using ECL (Tanon, Shanghai, China). The primary antibodies were as follows: TRIM25 (Cell Signaling technology, 1:1000 dilution, #13773), DDX5 (Cell Signaling technology (Danvers, MA, USA), 1:1000 dilution, #4387), YBX1 (ABclonal (Woburn, MA, USA), 1:2000 dilution, A7704), DHX9 (Abcam (Cambridge, UK), 1:1000 dilution, ab26271). Loading control antibodies are as follows: GAPDH (ABclonal, 1:2000 dilution, A19056), Vinculin (SantaCruz (Santa Cruz, CA, USA),1:1000 dilution, V284), H3 (Cell Signaling technology, 1:1000 dilution, #4499). 

### 4.4. Immunohistochemical Staining (IHC)

TRIM25 expression level in PCa tissue was analyzed by IHC. Briefly, the tissue microarray (Taibosi Biotechnology Co., Ltd., Xian, China) was incubated with TRIM25 primary antibody. After washing, the tissue chip was incubated with the HRP-labeled secondary antibodies, and then stained with the avidin–biotin peroxidase complex (ABC) method according to the protocol of manufacturer. Images were obtained using a large field macro magnification microscope (Zeiss, Axio Zoom.V16, Oberkochen, Germany).

### 4.5. Immunoprecipitation 

Cells were lysed with IP buffer (25 mM Tris-HCl pH 8.0, 1 mM DTT, 0.5% NP-40, 150 mM NaCl, 1 mM EDTA), and the harvested supernatant was incubated with Flag-M2 magic beads (Lot:TJ276517, Thermo Fisher, Waltham, MA, USA). The immune precipitates were then washed three times with IP buffer. The beads were finally boiled in 2 × SDS loading buffer. 

Other detailed experimental methods on cell biology and molecular biology are provided in the Supplementary Materials and Methods.

### 4.6. GC-MS

Sample preparation for GC–MS analysis was operated as follows: cells were harvested via scraping with 500 μL of pre-cooled methanol containing tridecanoic acid as the internal standard (10 μg/mL). The homogenate was further mixed through a vortex for 30 s and centrifuged at 15,000 *g* for 15 min at 4 °C. Then, 450 μL of the supernatant was isolated and dried by a rotating vacuum. Next, 50 μL of methoxyamine (in pyridine at concentration of 20 mg/mL) was applied to dissolve the residue, which was further re-dissolved via vortex for 5 min. The mixture was then incubated at 37 °C for 90 min. Subsequently, 40 μL of MSTFA was also used for the incubation at 37 °C for 60 min. The supernatant after centrifugation was transferred into the glass sampling vials for further GC–MS analysis.

For metabolic flux analysis, 40 μL of BSTFA was applied instead of MSTFA, the mixture was then incubated at 55 °C for 90 min. The following steps were carried out: Sample analysis was conducted upon a QP2010 plus series GC–MS system (Shimadzu, Kyoto, Japan) GC–MS separation was conducted using a DB-5ms fused-silica capillary column (30 m × 0.25 mm × 0.25 μm, Agilent Technology, Santa Clara, CA, USA). Pure helium was used as carrier gas. Injection volume was set as 1 μL with a split ratio of 10:1. The GC gradient used was as follows: 80 °C for 1 min, then increased to 210 °C at a rate of 30 °C/min, and further to 320 °C at 20 °C/min, and held for 4 min. The parameters for MS acquisition were set as follows: ionization voltage for electron impact was applied as 70 eV. Mass scan range was from 45 to 600 Da. The temperature of the ion source was 230 °C, while that of the quadrupole temperature was 150 °C.

The raw data were imported into GC–MS solution software (Shimadzu, Kyoto, Japan). The parameters for peak integration were set as follows: slope, 20/min; FWHM, 3 s; smoothing method, Savitzky–Golay; drift, 0/min; RT window (target), 5%; band width, 0.1 min. The generated peaks were normalized with the internal standard.

### 4.7. Statistical Analysis

GraphPad Prism 7.0 (GraphPad Software, Inc., San Diego, CA, USA) was used for statistical analysis. Data are presented as the mean  ±  SD to show the differences among groups. Significant differences are indicated based on asterisks (*: *p * <  0.05; **: *p*  <  0.01; ***: *p*  <  0.001).

## Figures and Tables

**Table 1:** The top 10 ranked genes and their AUC values.

Rank	Gene	AUC	Rank	Gene	AUC
1	MYC proto-oncogene (MYC)	0.73	6	EPH receptor A4 (EPHA4)	0.58
2	Cluster of differentiation-44 (CD44)	0.63	7	B-cell linker protein (BLNK)	0.84
3	Cyclin dependent kinase 6 (CDK6)	0.78	8	Phosphatase and tensin homolog (PTEN)	0.50
4	Wnt family member 5A (WNT5A)	0.80	9	RAN binding protein 2 (RANBP2)	0.85
5	Albumin (ALB)	0.76	10	Mechanistic target of rapamycin kinase (MTOR)	0.76

AUC: The area under the receiver operating characteristic curve. The top-ranked genes were selected by DMRG based on the topology properties of W-K-DN.

## Data Availability

The data presented in this study are openly available in GitHub at https://github.com/DLUT-datas/DMRG (accessed on 28 July 2022).

## Supplementary Materials

The following supporting information can be downloaded at: https://www.mdpi.com/article/10.3390/ijms23169325/s1.
