# Supplementary material for: Identification and Validation of TRIM25 as a Glucose Metabolism Regulator in Prostate Cancer
Source: Int J Mol Sci. 2022 Aug 19;23(16):9325. doi: 10.3390/ijms23169325 (PMC9408812; doi:10.3390/ijms23169325)
Supplement: Supplementary file 1 [file ijms-23-09325-s001.zip › ijms-1825475-supplementary.pdf]

Supporting Information

Identification and validation of TRIM25 as a glucose metabolism regulator in prostate cancer

Chao Li 1,2,†, Peng Dou 2,†,Xin Lu 2, Pengwei Guan 2, Zhikun Lin 2, Yanyan Zhou 2, Xin Lu 2,3,*, Xiaohui Lin 1 and Guowang Xu 2,3,*

1 School of Computer Science and Technology, Dalian University of Technology, Dalian 116024, China; lizimu@mail.dlut.edu.cn (C.L.); datas@dlut.edu.cn (X.L.)

2 CAS Key Laboratory of Separation Science for Analytical Chemistry, Dalian Institute of Chemical Physics, Chinese Academy of Sciences, Dalian 116023, China; doup@dicp.ac.cn (P.D.); luxin722@dicp.ac.cn (X.L.); guanpw2018@dicp.ac.cn (P.G.); linzk@dicp.ac.cn (Z.L.); zyy520@dicp.ac.cn (Y.Z.)

3 Liaoning Province Key Laboratory of Metabolomics, Dalian 116023, China

* Correspondence: luxin001@dicp.ac.cn (X.L.); xugw@dicp.ac.cn (G.X.)

† These authors contributed equally to this work.

Contents

Supplementary Materials and Methods S-3

1. SiRNA-mediated TRIM25 knockdown S-3

2. Lentiviral transduction S-3

3. Cell proliferation assay S-3

4. RNA-seq S-4

5. RT-qPCR S-5

6. Cell fractionation S-5

7. ATP assay S-6

8. RNA-Immunoprecipitation S-6

9. Protein profiling by LC-MS S-7

References S-8

Figure S1 Kaplan-Meier plots of patients with differently expressed genes (PRPF31, ISYNA1, TRIM25) obtained by DMRG. S-9

Figure S2 LDH-IN inhibit LDH activity to reduce lactate. S-9

Figure S3. Analysis of the interaction between DDX5 and TIRM25 truncator. S-10

Table S1. The 22 genes differentially expressed and related to PCa S-11

Table S2. The differential expression analysis of TRIM-family. S-122

Table S3. The sequence information of the knockdown siRNA of TRIM25. .... S-133

Table S4. Primer sequences for glucose metablism related genes S-13

Supplementary Materials and Methods

1. SiRNA-mediated TRIM25 knockdown

The knockdown of TRIM25 was achieved by transiently transfecting cells with siRNA targeting TRIM25. Briefly, cells were seeded to reach 70-80% confluence by the next day, Lipofectamine 2000 (Invitrogen) and siRNA were mixed with opti-medium respectively, and after standing at room temperature for 5 min, the two part mixtures were left to stand for 10 min, after which the mixture was added drop by drop to the cells. SiRNA of TRIM25 was synthesized at GenePharma (Shanghai, China). A total of 3 sequences are provided in Table S3, SiTRIM25 1# and SiTRIM25 2# were used in Figure 2A, and SiTRIM25 3# was used in Figure 3D.

2. Lentiviral transduction

Human TRIM25 Lentiviral Vector was constructed (pLVSIN, Takara) and was used to overexpress TRIM25 in PC3 cells. In the control group (Con) pLVSIN-empty vector was used to package the virus and infect the cells. Briefly, HEK293T cell was co-transfected with vesicular stomatitis virus glycoprotein (pLP-VSV-G) and pCMV-GAG-pol for 24 hr, after that, the fresh culture medium was used. Viral particles were obtained from HEK-293T packaging cell lines, The recombinant lentiviral particles of TRIM25 and the control in the viral supernatants were collected at 48 and 72 hr. Cells were infected once with viral supernatants, 1 μg/ml puromycin was used to select cells.

3. Cell proliferation assay

Cell proliferation was analyzed by Cell counting kit-8 (CCK-8, MCE) with operating manual. Cells were inoculated in 96-well plates, CCK-8 was mixed with cell culture medium at a ratio of 1:9, the cell culture medium was removed, 100 μL of

culture medium with CCK-8 was added to each well, and the cells were incubated in a cell incubator for 2 hr at 37°C. Afterward, the 96-well plates were assayed under 450 nm excitation light in a Molecular Devices (SpectraMax 190), and the values were counted. The results were plotted as mean±SD for three repeats in each condition.

4. RNA-seq

To identify TRIM25 regulated gene expression, we performed RNA-seq of PC-3 cells with overexpressed TRIM25 (n=3). Briefly, for RNA-seq Library Preparation, firstly, mRNA was isolated from total RNA using polyA selection, then cDNA was created, subsequently, cDNA was amplified and purified for cluster generation. Total RNA was isolated by using a TRIzol total RNA extraction kit (TIANGEN, Cat.No.DP424), which yielded > 2 μg of total RNA per sample. RNA quality was examined by 0.8% agarose gel electrophoresis and spectrophotometry. High-quality RNA with a 260/280 absorbance ratio of 1.8-2.2 was used for library construction and sequencing. Illumina HiSeq library construction was performed according to the manufacturer's instructions (Illumina, USA). Oligo-dT primers were used to transverse mRNA to obtain cDNA (APExBIO, Cat. No. K1159). cDNA for the synthesis of the second chain of cDNA was amplified, and cDNA products were purified by magnetic beads. After library construction, library fragments were enriched by PCR amplification and selected according to a fragment size of 350-550 bp. The library was quality-assessed using an Agilent 2100 Bioanalyzer (Agilent, USA) and sequenced using the Illumina NovaSeq 6000 sequencing platform (Paired end150) to generate raw reads.

Finally, bioinformatics analysis of the obtained sequences was performed. The raw paired-end fastq reads were filtered by TrimGaloreto to discard the adapters and

low quality bases via calling the Cutadapt tool. The clean reads obtained were then aligned to the mm10/hg19 mouse/human genome using HISAT2 followed by reference genome-guided transcriptome assembly and gene expression quantification using StringTie. Differentially expressed genes (DEGs) were identified by DEseq2 (for sample with replications) or edgeR (for sample with no replication) with a cut-off value of log2|fold-change|>1 and p-adjust <0.05. The related code and transcriptome data are available at https://github.com/DLUT-datas/DMRG..

5. RT-qPCR

RNA samples were reversely transcribed into cDNAs using PrimeScriptTMRT Master Mix (TaKaRa, Japan). cDNA was detected by real-time PCR analyses using LightCycler 480 SYBR Green I Master Mix (Roche) with a LightCycler 480 Instrument (Roche). Relative expression level quantifications were calculated by ΔΔCt method and normalized respect to GAPDH. Results were presented as fold change over the control group. Table S4 shows the primer sequences for glucose metabolism related genes.

6. Cell fractionation

Cell fractionation was performed as described[1], briefly, 10-cm dish cells were harvest, and then incubated in buffer (10mM Hepes, pH 7.9; 1.5 mM $MgCl_2$; 10 mM KCl; 0.34 M sucrose; 10% (vol/vol) glycerol; 0.1% Triton X-100; 1 mM DTT; 1:1,000 protease inhibitor mixture) on ice for 5 min, then 500 g centrifuged for 5 min. Next, supernatant was collected and centrifuged at 15000 g for 15 min, after that, supernatant and pellet were collected for use. Nuclear was lysed by nuclear lysis buffer (2 mM EGTA) and then centrifuged at 500 g for 5 min, the pellet was sonicated

by non-contract ultrasonic crushing at 100 HZ puzzled 5s. All the components were mixed with a loading buffer.

7. ATP assay

ATP assay was performed according to ATP Assay Kit (S0026 Beyotime) protocol, briefly, 6-well plate cell growth up to 80% confluence. Next, cells were lysed and centrifuge at 12000 rpm for 15 min at 4℃. Supernatant was removed into a new tube and kept on ice. Diluted the ATP standard solution of different concentrations and added the diluted working solution in a 96-well plate. Then, ATP detection (ATP detection reagent diluent: ATP detection reagent 5:1) solution was added into diluted working solution and cell lysis supernatant. Finally, the microplate reader detected and calculated ATP content.

8. RNA-Immunoprecipitation

RNA-Immunoprecipitation was performed according to protocol[2], briefly, cell lysis was performed with immunoprecipitation (IP) buffer: 20 mM Tris-HCl pH 8.0; 200 mM NaCl; 1 mM EGTA; 1 mM EDTA; 0.5% Triton X-100; 0.4 U/μL RNasin, protease inhibitor cocktail (MCE, China). Cell lysis was immunoprecipitated using Flag-M2 beads for overnight at 4℃, after that, beads were washed with PBS. Then, remove supernatant completely from the beads and resuspend beads with 50 μL Proteinase K buffer. 2 μL of Proteinase K was added, and incubated at 50℃. Centrifuged beads and transferred the supernatant to a fresh tube. And then, add 1 μL of glycogen and 1 ml of TRizol to extract RNA. Finally, RNA was detected according to PCR protocol.

9. Protein profiling by LC-MS

For trypsin digestion within the gel, gels fractions were decontaminated in 50 mM NH_4HCO_3 in 50% acetonitrile (v/v) until they were clear. The pieces of gel were desiccated with 100 μL of 100% of acetonitrile for 5 min, the solution was then removed, and the gel pieces were resuspended in 10 mM of dithiothreitol and cultured at 37°C for 60 min. The slices were again desiccated in 100% of acetonitrile, the liquid was removed, and gel pieces were rehydrated with 55 mM iodoacetamide. Samples were incubated at room temperature and left in dark for 45 min. The gel slices were treated with 50 mM NH_4HCO_3 and dried with 100% acetonitrile. The gel slices were rehydrated with 10 ng/μL of trypsin digestion in 50 mM NH_4HCO_3 and resuspended on ice for 1 h. Digestion was performed with trypsin at 37°C overnight. Peptides were extracted with 50% acetonitrile/5% formic acid, followed by 100% acetonitrile. Peptides were dried and redissolved in 2% acetonitrile/0.1% formic acid (buffer A).

Above tryptic peptides solution was loaded directly onto a homemade reversed-phase analytical column (15 cm long, 75 μm diameter). The gradient was comprised of an increase from 6% to 35% buffer B (0.1% formic acid in 98% acetonitrile) in 16 min, 35% to 80% in 8 min and kept for 3 min, a constant flow rate of 550 nL/min was used on an EASY-nLC 1000 UPLC system.

The used detector was Q ExactiveTM Plus (Thermo, Waltham, USA) with the nanoelectrospray ionization (NSI) source. The ion source voltage was 2.0 kV, m/z scan range was from 350 to 1800 (full scan), and intact peptides were detected by the Orbitrap at a resolution of 70,000. Peptides were then selected for MS/MS using normalized collision energy (NCE) set at 28 and the fragments were detected in the Orbitrap at a resolution of 17,500. The data acquisition mode was a data-dependent

scanning (DDA) procedure, i.e., the top 20 peptide parent ions with the highest signal intensity were selected after the primary scan. To improve the effective utilization of the mass spectrum, the automatic gain control (AGC) was set to 5e4, the signal threshold was set to 5e3 ions/s, the maximum injection time was set to 200 ms, and the dynamic exclusion time of the tandem mass spectrometry scan was set to 15.0 s to reduce the number of parent ion of repeated scans.

References

1. Yang, L.; Gal, J.; Chen, J.; Zhu, H. Self-assembled FUS binds active chromatin and regulates gene transcription. *Proc Natl Acad Sci U S A* **2014**, 111, 17809-17814, doi:10.1073/pnas.1414004111.

2. Bierhoff, H. Analysis of lncRNA-Protein Interactions by RNA-Protein Pull-Down Assays and RNA Immunoprecipitation (RIP). *Methods Mol Biol* **2018**, 1686, 241-250, doi:10.1007/978-1-4939-7371-2_17.

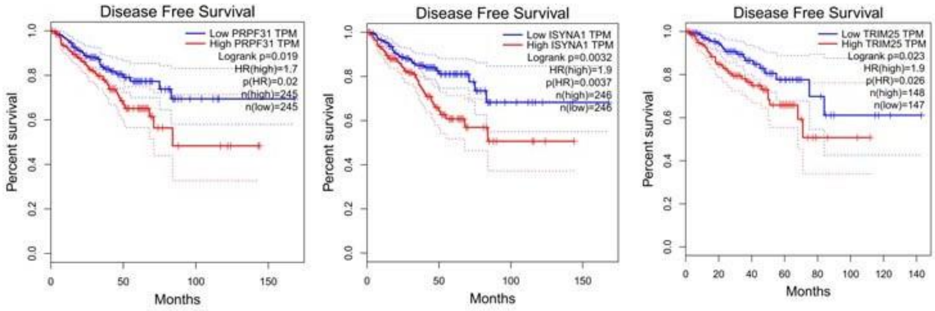

Figure S1. Kaplan-Meier plots of patients with differently expressed genes (PRPF31, ISYNA1, TRIM25) obtained by DMRG

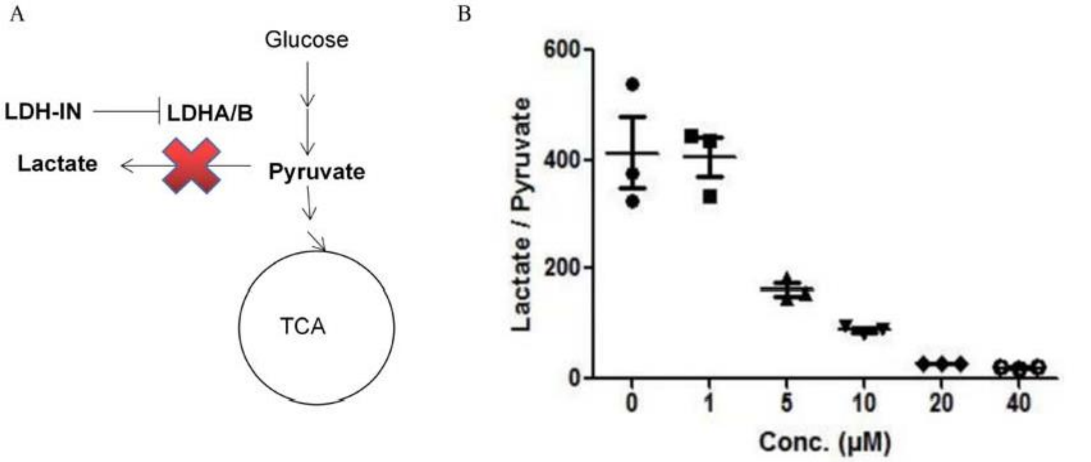

Figure S2. LDH-IN inhibits LDH activity to reduce lactate. A. Working model of LDH-IN target. B. The effects of different concentration of LDH-IN on pyruvate/lactic acid transformation in PC3 cells.

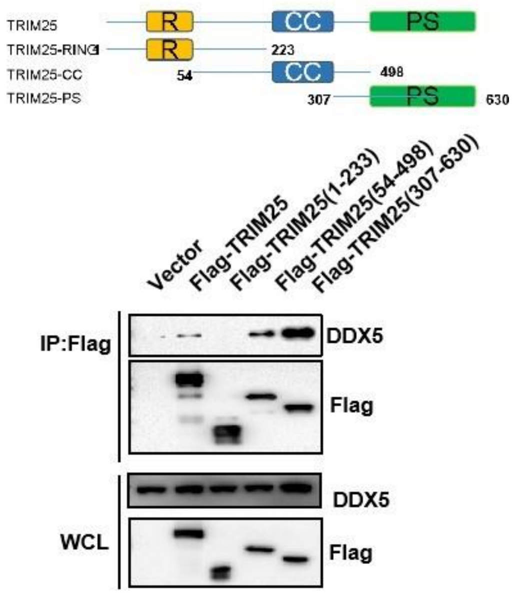

Figure S3. Analysis of the interaction between DDX5 and TIRM25 truncator. Three truncators were constructed containing the TRIM25 functional domain, specifically a RING-containing domain at amino acids 1-233, a CC-containing domain at amino acids 54-498, and a PS-containing domain at amino acids 307-630. The truncators of TRIM25 were further analyzed by immunoprecipitation to interact with DDX5.

Table S1. The 22 genes differentially expressed and related to PCa.

Gene symbol	Gene name	*p* value
NR3C1	Nuclear receptor subfamily 3 group C member 1	6.44×10^{-3}
NUP43	Nucleoporin 43	8.68×10^{-4}
MCC	MCC regulator of WNT signaling pathway	8.68×10^{-4}
ASB2	Ankyrin repeat and SOCS box containing 2	4.02×10^{-3}
S100B	S100 calcium binding protein B	3.08×10^{-3}
SETDB1	SET domain bifurcated histone lysine methyltransferase 1	8.06×10^{-3}
CD40	Cluster of differentiation-40	2.05×10^{-3}
RCBTB2	RCC1 and BTB domain containing protein 2	4.02×10^{-3}
FLNA	Filamin A	6.70×10^{-3}
CIB2	Calcium and integrin binding family member 2	4.29×10^{-3}
TLR3	Toll like receptor 3	4.02×10^{-3}
STAT1	Signal transducer and activator of transcription 1	1.45×10^{-2}
ISYNA1	Inositol-3-phosphate synthase 1	7.58×10^{-3}
TTC28	Tetratricopeptide repeat domain 28	4.89×10^{-2}
RALY	RALY heterogeneous nuclear ribonucleoprotein	1.82×10^{-2}
ABCA4	ATP binding cassette subfamily A member 4	4.64×10^{-2}
WWP2	WW domain containing E3 ubiquitin protein ligase 2	3.82×10^{-2}
PRPF31	Pre-mRNA processing factor 31	2.96×10^{-2}
NUP153	Nucleoporin 153	3.46×10^{-2}
LRIG3	Leucine rich repeats and immunoglobulin like domains 3	4.02×10^{-2}
TRIM25	Tripartite motif containing 25	3.12×10^{-2}
MUL1	Mitochondrial E3 ubiquitin protein ligase 1	4.89×10^{-2}

These genes are the neighbor of the node RANBP2 in the W-K-DN. These genes are selected for survival analysis with the TCGA PCa dataset. The statistical significance *p* value was obtained by T-test.

Table S2. The differential expression analysis of TRIM-family.

Gene symbol	*p* value	Gene symbol	*p* value	Gene symbol	*p* value
TRIM33	3.01×10^{-6}	TRIM14	1.35×10^{-1}	TRIM52	4.27×10^{-1}
TRIM37	9.74×10^{-6}	TRIM43B	2.52×10^{-1}	TRIM67	2.44×10^{-1}
TRIM36	1.11×10^{-4}	TRIM17	1.06×10^{-1}	TRIM22	1.64×10^{-1}
TRIM59	2.33×10^{-4}	TRIM73	3.70×10^{-1}	TRIM8	2.98×10^{-1}
TRIM72	1.80×10^{-3}	TRIM65	1.70×10^{-1}	TRIM16	2.20×10^{-1}
TRIM29	3.25×10^{-5}	TRIM28	6.80×10^{-2}	TRIM47	8.13×10^{-1}
TRIM9	4.88×10^{-3}	TRIM11	$1.77E \times 10^{-1}$	TRIM49	8.20×10^{-1}
TRIM44	6.70×10^{-3}	TRIM71	$3.79E \times 10^{-1}$	TRIM13	7.18×10^{-1}
TRIM24	6.94×10^{-4}	TRIM26	5.57×10^{-1}	TRIM6-TRIM34	3.38×10^{-1}
TRIM27	1.30×10^{-2}	TRIM41	1.97×10^{-1}	TRIM35	5.29×10^{-1}
TRIM5	5.92×10^{-2}	TRIM49L1	2.97×10^{-1}	TRIM21	5.43×10^{-1}
TRIM54	8.10×10^{-2}	TRIM61	2.61×10^{-1}	TRIM60	5.84×10^{-1}
TRIM68	4.74×10^{-4}	TRIM66	2.20×10^{-1}	TRIM69	4.03×10^{-1}
TRIM2	5.92×10^{-2}	TRIM62	2.70×10^{-1}	TRIM63	8.29×10^{-1}
TRIM39	2.96×10^{-2}	TRIM32	9.92×10^{-1}	TRIM58	4.04×10^{-1}
TRIM3	4.02×10^{-2}	TRIM23	7.97×10^{-1}	TRIM4	3.27×10^{-1}
TRIM50	9.49×10^{-2}	TRIM7	5.99×10^{-1}	TRIM53P	8.93×10^{-1}
TRIM25	3.12×10^{-2}	TRIM45	5.99×10^{-1}	TRIM56	9.10×10^{-1}
TRIM16L	5.92×10^{-2}	TRIM38	8.61×10^{-1}	TRIM46	6.28×10^{-1}

The statistical significance *p* value was obtained by T-test.

Table S3. The sequence information of the knockdown siRNA of TRIM25.

Gene	Primer and siRNA sequences
NC	UUCUCCUGAACGUGUCACGUTT
SiTRIM25 1#	GGCACAAACUAACUGUCAUTT
SiTRIM25 2#	CCUCGACAAGGAAGAUAAATT
SiTRIM25 3#	GCUCCUGGAGUAUUACAUUTT

Table S4. Primer sequences for glucose metabolism related genes.

Gene	Primer sequences
CS F	TGCTTCCTCCACGAATTTGAAA
CS R	CCACCATACATCATGTCCACAG
DLD F	GAAATGTCCGAAGTTCGCTTGA
DLDR	TCAGCTTTCGTAGCAGTGACT
DLST F	GAACTGCCCTCTAGGGAGAC
DLST R	AACCTTCCTGCTGTTAGGGTA
FH F	GGAGGTGTGACAGAACGCAT
FH R	CATCTGCTGCCTTCATTATTGC
IDH1 F	AGAAGCATAATGTTGGCGTCA
IDH1 R	CGTATGGTGCCATTTGGTGATT
IDH2 F	CCCGTATTATCTGGCAGTTCATC
IDH2 R	ATCAGTCTGGTCACGGTTTGG
MDH1 F	GGTGCAGCCTTAGATAAATACGC
MDH1 R	AGTCAAGCAACTGAAGTTCTCC
MDH2 F	TCGGCCCAGAACAATGCTAAA
MDH2 R	GCGGCTTTGGTCTCGATGT
OGDH F	GGCTTCCCAGACTGTTAAGAC
OGDH R	GCAGAATAGCACCGAATCTGTTG
PC F	ACAGAGGTGAGATTGCCATCC

PC R	CACTGCATCTACGTTGTTCTCC
PDK2 F	ATGAAAGAGATCAACCTGCTTCC
PDK2 R	GGCTCTGGACATACCAGCTC
PDK3 F	CGCTCTCCATCAAACAATTCCT
PDK3 R	CCACTGAAGGGCGGTTAAGTA
SDHA F	CAAACAGGAACCCGAGGTTTT
SDHA R	CAGCTTGGTAACACATGCTGTAT
SDHB F	ACAGCTCCCCGTATCAAGAAA
SDHB R	GCATGATCTTCGGAAGGTCAA
SDHD F	ATTTCTTCAGGACCGACCTATCC
SDHD R	CAGCCTTGGAGCCAGAATG
